# SGR-YOLO: a method for detecting seed germination rate in wild rice

**DOI:** 10.3389/fpls.2023.1305081

**Published:** 2024-01-23

**Authors:** Qiong Yao, Xiaoming Zheng, Guomin Zhou, Jianhua Zhang

**Affiliations:** ^1^ College of Agriculture, Henan University, Zhengzhou, China; ^2^ National Academy of Southern Breeding, Chinese Academy of Agricultural Sciences, Sanya, China; ^3^ Institute of Crop Sciences, Chinese Academy of Agricultural Sciences, Beijing, China; ^4^ Agricultural Information Institute of Chinese Academy of Agricultural Sciences/National Agricultural Science Data Center, Beijing, China

**Keywords:** wild rice, germination detection, deep learning, SGR-YOLO, BiFPN

## Abstract

Seed germination rate is one of the important indicators in measuring seed quality and seed germination ability, and it is also an important basis for evaluating the growth potential and planting effect of seeds. In order to detect seed germination rates more efficiently and achieve automated detection, this study focuses on wild rice as the research subject. A novel method for detecting wild rice germination rates is introduced, leveraging the SGR-YOLO model through deep learning techniques. The SGR-YOLO model incorporates the convolutional block attention module (efficient channel attention (ECA)) in the Backbone, adopts the structure of bi-directional feature pyramid network (BiFPN) in the Neck part, adopts the generalized intersection over union (GIOU) function as the loss function in the Prediction part, and adopts the GIOU function as the loss function by setting the weighting coefficient to accelerate the detection of the seed germination rate. In the Prediction part, the GIOU function is used as the loss function to accelerate the learning of high-confidence targets by setting the weight coefficients to further improve the detection accuracy of seed germination rate. The results showed that the accuracy of the SGR-YOLO model for wild rice seed germination discrimination was 94% for the hydroponic box and 98.2% for the Petri dish. The errors of germination potential, germination index, and average germination days detected by SGR-YOLO using the manual statistics were 0.4%, 2.2, and 0.9 days, respectively, in the hydroponic box and 0.5%, 0.5, and 0.24 days, respectively, in the Petri dish. The above results showed that the SGR-YOLO model can realize the rapid detection of germination rate, germination potential, germination index, and average germination days of wild rice seeds, which can provide a reference for the rapid detection of crop seed germination rate.

## 1 Introduction

Wild rice is a natural gene pool of rice germplasm resources, and under a long-term natural environment, it has accumulated many excellent genes that cultivated rice does not possess, and has numerous specific traits that can be utilized for breeding and biotechnology of cultivated rice, which is conducive to the genetic improvement of cultivated rice (Zhong et al., 2000). Wild rice is important for breeding because it possesses many valuable genetic traits and stress tolerance, such as drought tolerance, salt tolerance, and resistance to pests and diseases (Quan et al., 2018; Yang et al., 2022). These traits can help to improve cultivated rice varieties for better yield and adaptability. The study of genome and genetic variation in wild rice can help to improve the yield and quality of modern crops (Tanksley and McCouch, 1997; Tian et al., 2006).

The germination rate of wild rice seeds is very low and untidy, which causes great difficulties in the conservation, identification, and utilization studies of wild rice (Zhao et al., 1998). The germination rate of wild rice seeds is one of the most important indicators of seed quality. Seeds with high germination rate germinate quickly in the field and have a high resistance to adversity; seeds with low germination rate germinate slowly in the field, have irregular seedling emergence, and are susceptible to the effects of the growing environment, which may cause a reduction in the yield of agricultural products (Li and Qin, 1998). Wild rice seed germination is usually assessed manually by counting the number of embryonic sheaths visible in the Petri dish. Seeds are generally considered to have germinated when the length of the embryonic sheath exceeds 2 mm (Tobe et al., 2000). Seed germination labeling is performed by an experienced person, who labels seed categories by visually discerning seed radicle length and germ length. Traditional germination detection is through human eye observation. The germination of seeds germinated for 7 days is measured through statistical judgment (Cheng et al., 2002). The inspector needs to have a wealth of experience, as the repetitive germination rate detection is very cumbersome, time-consuming, laborious, and easy to introduce subjective errors, resulting in inconsistent statistical results between different people and poor reproducibility (Li, 2016). Therefore, there is a need for an objective, reproducible, rapid, and economically reliable method of determination.

In recent years, deep learning has developed rapidly and has been widely used in the field of agriculture, and many researchers have applied deep learning to germination detection of seeds (Yuan et al., 2016; Dang et al., 2020; Zhang et al., 2021). Joosen et al. (2010) chose a semiautomatic approach and designed a germinator to make a judgment of whether a seed has germinated or not by high-throughput scoring, which can handle many samples that may germinate under different environmental conditions. However, a good contrast between the radicle and seed coat is required, which may limit its use in several crops. Zhang et al. (2023) utilized the techniques of image segmentation, transformer encoder, small target detection layer, and CDIOU loss to improve the accuracy of detection and developed a convolutional neural network (YOLO-r) that can detect the germination status of rice seeds and automatically evaluate the total number of germinations. The average accuracy was 0.9539, and the average absolute error in predicting the germination rate mainly existed within 0.1. Bai et al. (2023) constructed DB-YOLOv5, a model for seed germination discrimination, by combining machine vision technology with deep learning methods for rapid detection of germination rate, germination potential, germination index, and average days to germination of wheat seeds and carried out testing experiments. The accuracy of the DB-YOLOv5 model for germination discrimination of wheat seeds was 98.5%. Although the above studies achieved good accuracy, they only considered one culture method and a specific period of time and did not take into account the situation of different culture methods and different days.

In this study, a germination discrimination model for wild rice seeds was developed based on a deep learning network model for wild rice seed germination detection. Two different culture methods, namely hydroponic box and Petri dish, were used to detect the seeds in conjunction with a 7-day continuous germination test of wild rice seeds. The SGR-YOLO model was constructed, and based on the YOLOv7 model, the parts of the model were improved by adding the efficient channel attention (ECA) attention mechanism module, the structure of the bi-directional feature pyramid network (BiFPN), and the generalized intersection over union (GIOU) loss function. This method is able to better recognize and evaluate the germination of wild rice seeds, thus providing a fast and accurate solution for the assessment of seed quality. It realizes the detection of the germination rate of wild rice seeds in different culture methods and the rapid detection of germination rate, germination potential, germination index, and average germination days of wild rice seeds. It provides a feasible method for intelligent detection of seed germination rate.

## 2 Materials and methods

### 2.1 Construction of wild rice seed germination dataset

#### 2.1.1 Wild rice seed material

The experimental materials for this study were wild rice numbers 843–1129, with 27 grains of each number, totaling 7,749 seeds, all of which were obtained from the Institute of Crop Science, Chinese Academy of Agricultural Sciences. There are phenotypic differences between different strains of wild rice seeds. **Table 1** lists some cereal seeds with different phenotypic characteristics, which vary greatly in length, shape, and color, including medium and short grain lengths; elongated, medium, and short thick shapes; light brown, brown, dark brown, and red colors; and awned versus awnless awns.

**Table 1:** Seeds with different phenotypic characteristics of seed grain.

Image	Grain length	Grain shape	Grain color	Awn
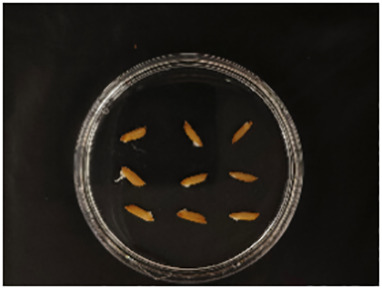	Medium	Slender	Brown	×
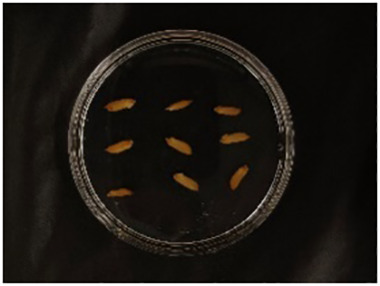	Medium	Medium	Brown	×
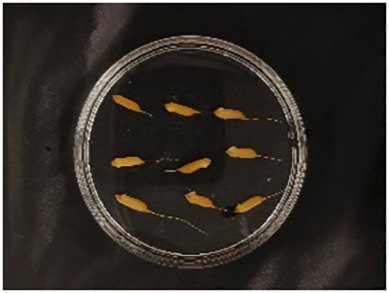	Medium	Medium	Light brown	√
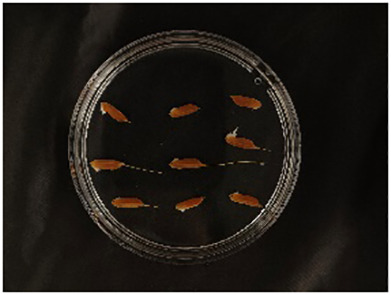	Medium	Medium	Red	√
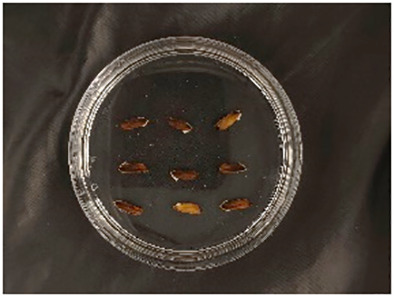	Short	Bold	Dark brown	×
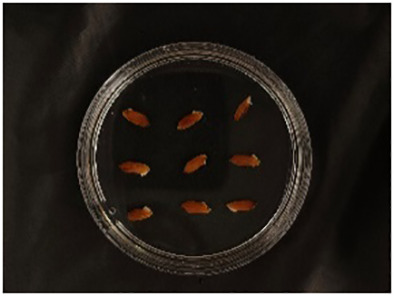	Short	Bold	Red	×
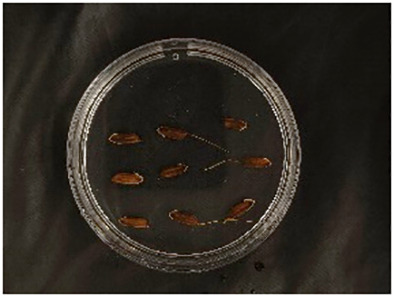	Medium	Medium	Dark brown	√
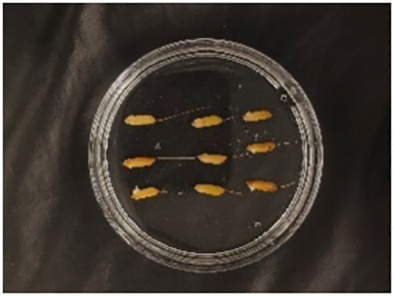	Short	Medium	Light brown	√
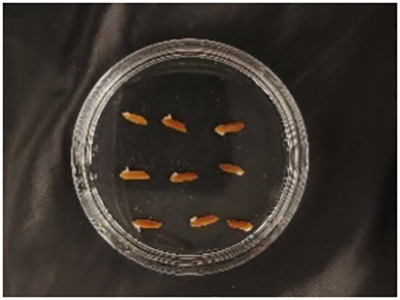	Medium	Slender	Red	×

#### 2.1.2 Image acquisition

Normally, seed germination is conducted using two methods—hydroponic box and Petri dish (Wang et al., 1996; Wang et al., 2002)—which can save test time and reduce contamination of seeds in the germination process. Therefore, the present study was conducted to test these two methods separately.

**Figure 1:**
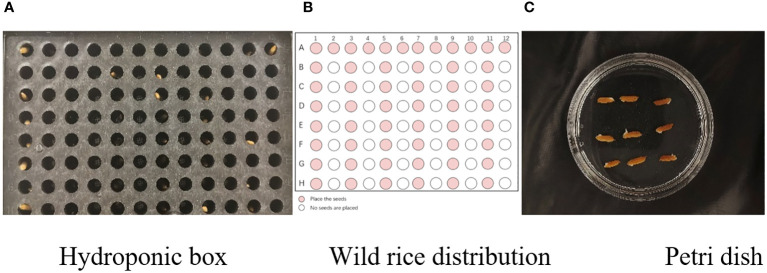
Wild rice placement. **(A)** Hydroponic box. **(B)** Wild rice distribution. **(C)** Petri dish.

In this experiment, seeds with full grains were baked in an oven at 40°C–45°C for 2–3 days, sterilized with 2%~3% sodium hypochlorite for 0.5 h, and soaked in an incubator at a constant temperature of 37°C, which was used to break seed dormancy (Lei et al., 2004). Next, the treated seeds were placed in Petri dishes and hydroponic boxes. For the Petri dish germination method, nine wild rice seeds of the same variety were placed in a single Petri dish (**Figure 1C**), and an appropriate amount of distilled water was added to each Petri dish in order to prevent water evaporation. The Petri dishes were then incubated at a constant temperature of 28°C. For the hydroponic box germination method, seeds were selected and transplanted into 96-well black hydroponic boxes containing fresh water (**Figure 1A**), and the light incubator conditions were set at 16,000 lx of light, 28°C, 12 h; darkness, 28°C, 12 h; and 75% humidity. To ensure that each seed could absorb nutrients evenly, seeds were placed according to varieties, with one variety in each row and another seed in the first row on the right side (as shown in **Figure 1B**) for a 7-day germination test.

The seeds were placed in Petri dishes and hydroponic boxes, with 1,123 seeds in Petri dishes and 6,626 seeds in hydroponic boxes, totaling 7,749 seeds.

**Figure 2:**
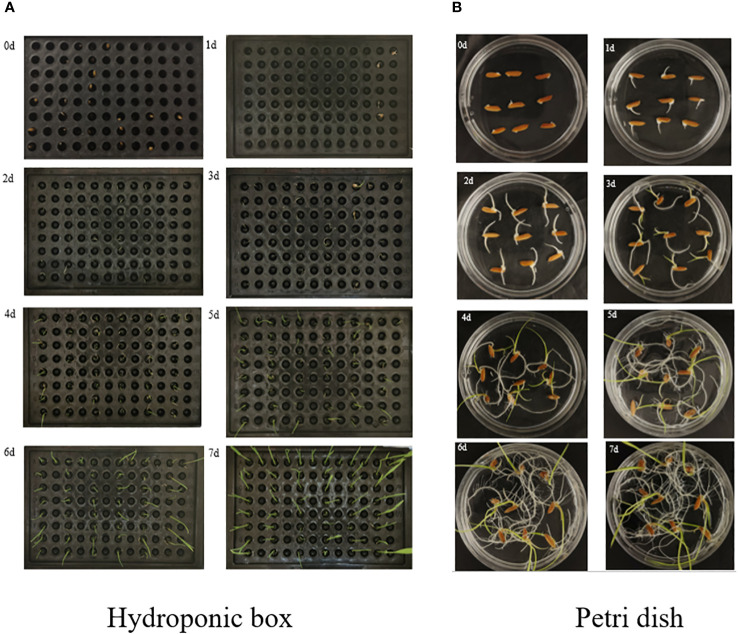
Capturing images. **(A)** Hydroponic box. **(B)** Petri dish.

In order to detect the whole process of seed germination, images were acquired from day 1 to day 7 by image acquisition performed on Petri dishes and hydroponic cassettes separately. The first image was taken immediately after the seeds were put into the incubator as the initial image (recorded as day 0), and the images were taken every 24 h for seven consecutive days, as shown in **Figure 2**. The image acquisition method was vertical shooting, the shooting time was 16:00–18:00, the original image size was 4,096 × 3,072, the acquisition device was a realme cell phone with a 50-megapixel camera and a 2-megapixel rear camera, the shooting process was set to two times digital zoom, and the image format was JPG. The photos taken were characterized by high quality and full color.

### 2.2 Constructing the dataset

For the two germination methods of hydroponic box and Petri dish, the data of the whole process of wild rice seed germination were collected, and the dataset of seed germination rate detection was formed. As shown in **Table 2**, in terms of days, on day 0, there were 310 frames; on day 1, there were 293 frames; on day 2, there were 292 frames; on day 3, there were 283 frames; on day 4, there were 281 frames; on day 5, there were 287 frames; on day 6, there were 284 frames; and on day 7, there were 280 frames. In terms of the culture method, there were 1,108 frames in hydroponic boxes and 1,202 frames in Petri dishes, totaling 2,310 frames.

**Table 2:** Sample size.

Days to germination	Hydroponic box image data volume/frame	Petri dish image data volume/frame	Aggregate/frame
0	154	156	310
1	137	156	293
2	135	157	292
3	135	148	283
4	136	145	281
5	141	146	287
6	135	149	284
7	135	145	280
Aggregate/frame	1,108	1,202	2,310

**Figure 3:**
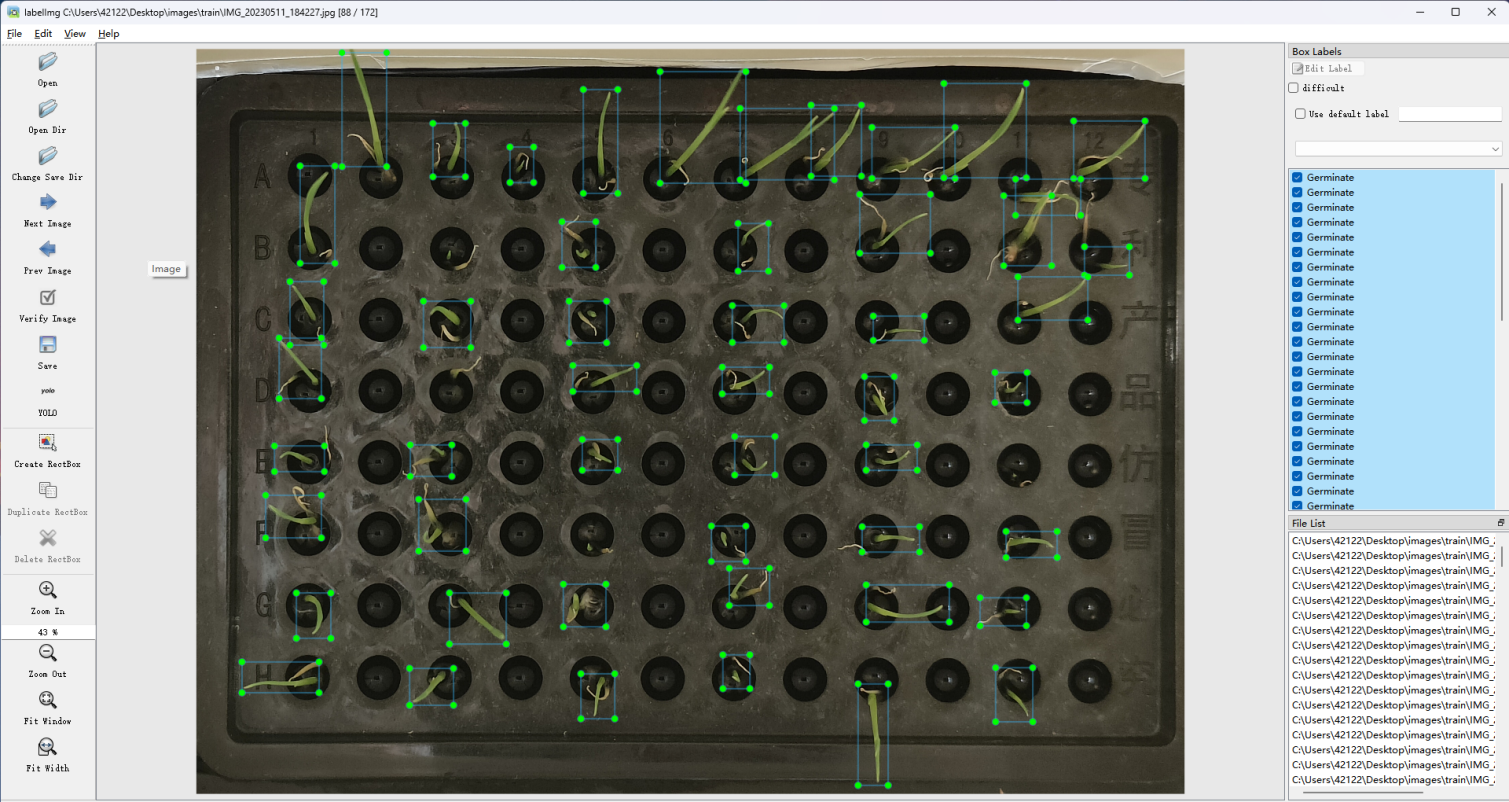
Labeling of germinated seeds.

The wild rice seeds in the captured images were labeled using the labelImg software, as shown in **Figure 3**, where the images were labeled using a rectangular box, with the seeds located in the middle of the rectangular box, and the position of the rectangular box was determined by the coordinates of its two diagonal corners. Different rectangular boxes were labeled in each image according to the location of the wild rice seeds.

The seeds in each image were labeled using labelImg software. The dataset was divided according to stratified sampling, and a total of 1,108 images were obtained from the hydroponic box, with the division ratio of training set:validation set:testing set = 7:2:1, which corresponded to the number of images collected as 776, 222, and 110, respectively. A total of 1,202 images were obtained from Petri dishes, and the division ratio was training set:validation set:test set = 7:2:1, which corresponded to the number of acquired images, as 841, 241, and 120, respectively.

### 2.3 Wild rice seed germination detection algorithm

#### 2.3.1 The SGR-YOLO network model

**Figure 4:**
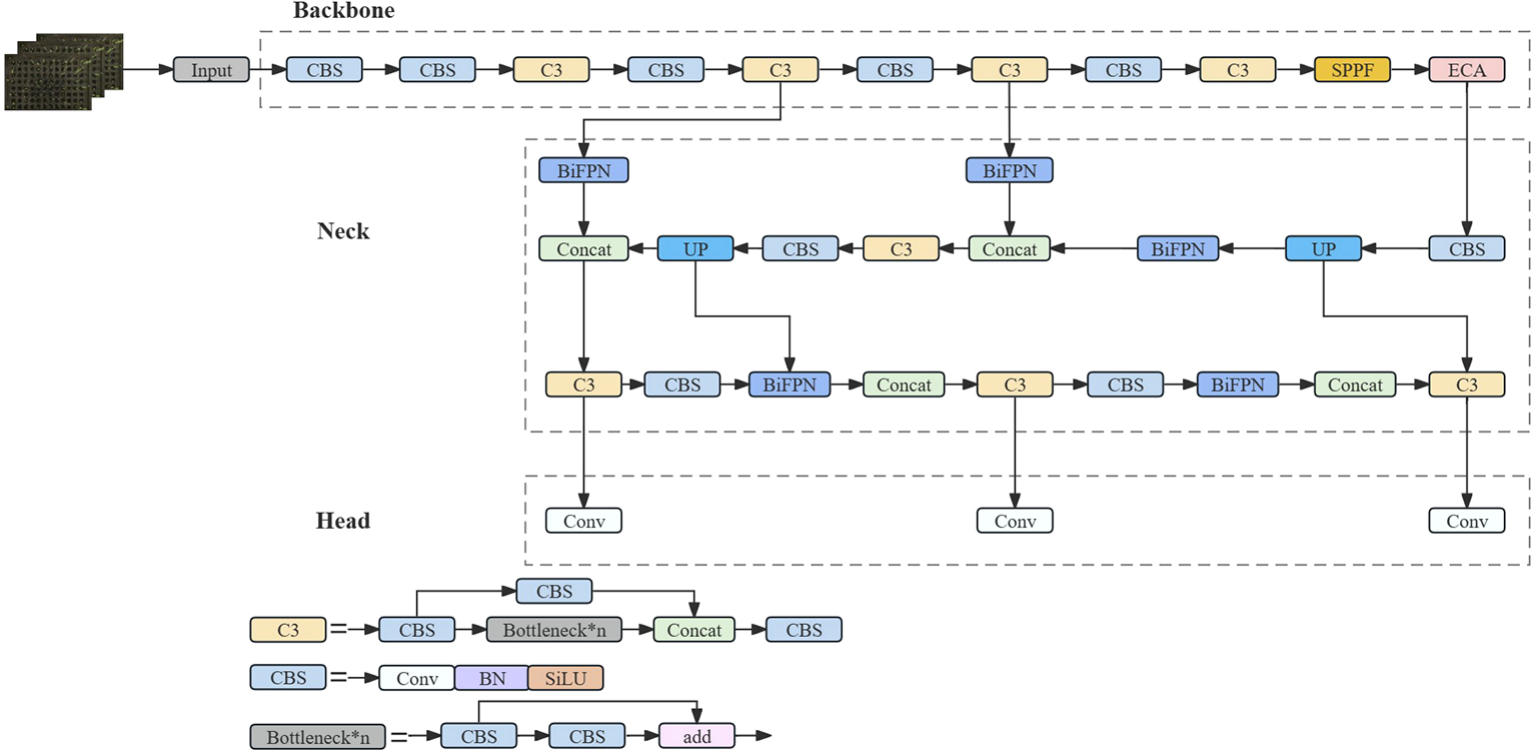
SGR-YOLO network structure diagram.

The small size of the individual wild rice seeds makes it much more difficult to discern germination problems. It shows different morphologies on different days, with dewy whites starting to appear on the first day and the first leaves starting to grow on the third day. During the growth and development of the seeds, the leaves overlapped and crossed over as the number of days changed, and the leaves were not uniform in size due to different growth rates. In a Petri dish, the buds and roots of the seeds grew at the same time, creating a complex background. In hydroponic boxes, each hole appeared as a reflection, thereby reducing the accuracy of the detection model. Therefore, there was a need to improve the thermal visualization, accuracy, and inference speed of the detection algorithm, and an improved feature extraction module was used. By optimizing the feature extraction capability and functionality in order to better capture the key features of the image, a more accurate and lightweight model structure can be obtained, and the network structure is shown in **Figure 4**.

The SGR-YOLO network structure adds the bi-directional feature pyramid network structure, the ECA attention mechanism module, and the GIOU loss function on the basis of YOLOv7. The small target detection layer was added to detect small targets. The network structure was based on the C3 module and SPPF as the backbone feature extraction network, and the feature pyramid was modified. The model mainly contains four parts: the input (Input), the backbone feature extraction network (Backbone), the neck network (Neck), and the head (Head). The image was sent to the Input after pre-processing operations, such as data enhancement of some of the data, and then sent to the network consisting of the C3 module and CBS module for feature extraction. Subsequently, the extracted features were fused in the Neck using Feature Pyramid Networks (FPNs) to obtain features of three sizes—large, medium, and small—combining the tensor splicing operation of Feature Fusion Networks with the weighted bi-directional feature pyramid BiFPN to target small targets to improve the feature fusion capability. Finally, the Head part generated the predicted category probability and location information of the target. In order to accelerate the convergence of the loss function of the predicted bounding box, the loss function GIOU was used, which effectively improved the performance of the model’s bounding box regression and improved the overall performance of the detection model in order to solve the problem of the detection needs of different environments.

#### 2.3.2 YOLOv7

YOLOv7 was proposed by Wang et al. (2022) in August 2022. YOLOv7 utilizes a single-stage detection method, which views the entire detection process as a regression problem. Compared to the traditional two-stage approach, YOLOv7 is faster and can maintain high accuracy in real-time application scenarios. The architecture of YOLOv7 consists of three parts: the Feature Extraction Network (Backbone), the Feature Fusion Layer (Neck), and the Prediction Layer (Head). The Backbone feature extraction network uses a powerful convolutional neural network to extract rich feature representations from the input image. The Neck feature fusion layer fuses feature maps at different scales for detection on targets of different sizes. The Head prediction layer outputs information such as the location, category, and confidence level of the target through multi-layer convolution and fully connected layers. Therefore, YOLOv7 has fast inference speed and excellent detection performance. It is suitable for real-time applications that require efficient object detection. In conclusion, YOLOv7 is a high-performance object detection model that can accurately and quickly detect a variety of targets.

#### 2.3.3 BiFPN

BiFPN (Tan et al., 2020) is known as the bi-directional feature pyramid network, proposed by Google. BiFPN is a bi-directional feature pyramid network utilizing the idea of bi-directional fusion. In target detection, a feature pyramid can improve the accuracy of target detection because it can combine features of different scales for inspection. BiFPN can realize multi-scale target detection with faster processing speed.

**Figure 5:**
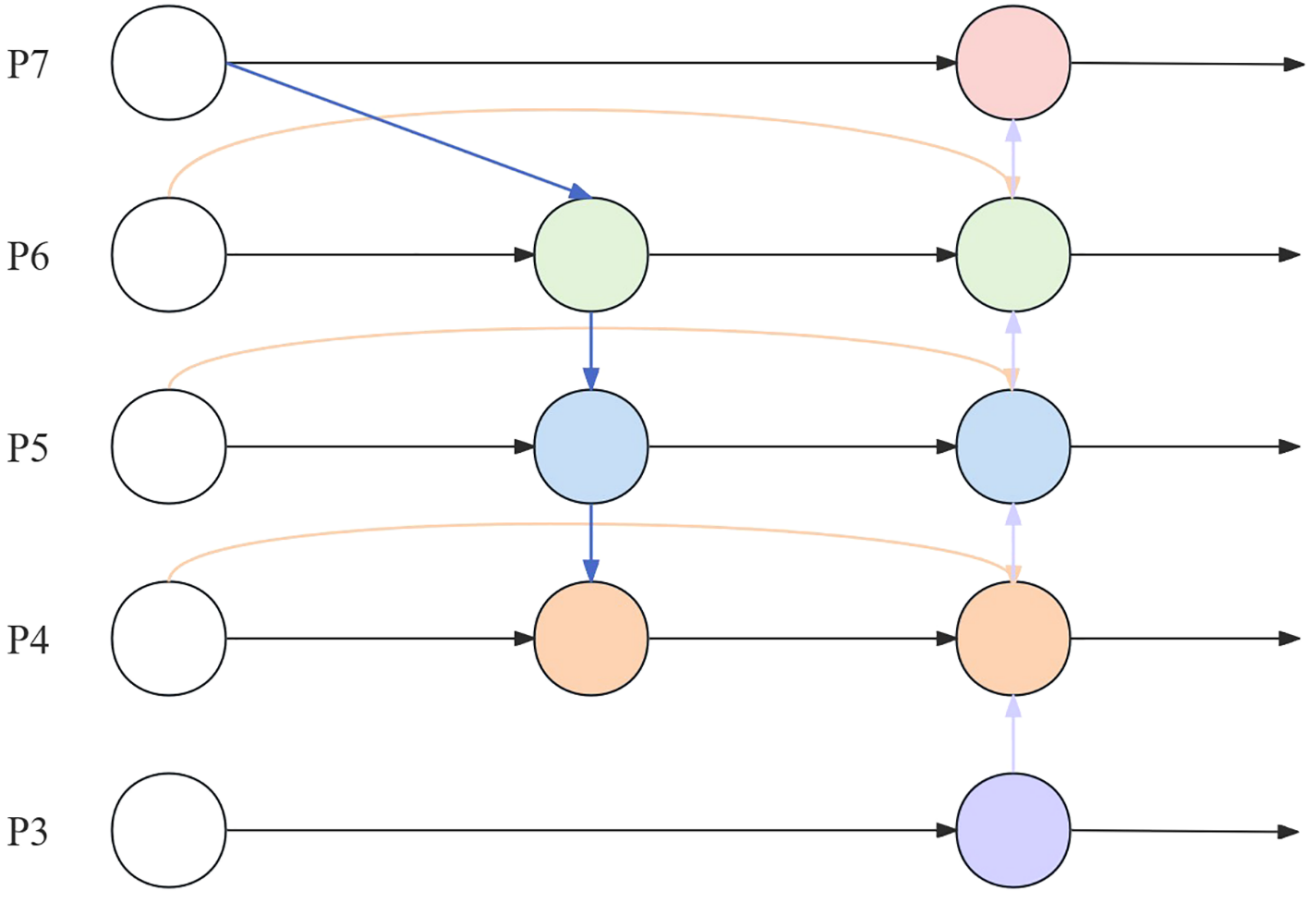
BiFPN structure. BiFPN, bi-directional feature pyramid network.

Neck uses an FPN + Path Aggregation Network (PAN) structure. This structure solves the problem of unidirectional information flow limitation of the traditional top-down FPN by adding a bottom-up path aggregation network through PAN. However, due to the dense target density of the wild rice germination detection task, the large number of targets in a single image and the large amount of computation lead to poor real-time performance when the model is applied to the wild rice germination detection task in different environments. In order to ensure the real-time performance of the wild rice germination detection task, BiFPN replaces FPN in Neck, as shown in **Figure 5**, with P7~P3 representing the layers where the nodes are located.

BiFPN introduces top-down and bottom-up bi-directional connections to realize multi-level information transfer and feedback. This can fully utilize the feature information between different layers, enabling the model to capture the details and contextual information of the object and improve the accuracy and robustness of target detection. BiFPN employs an effective feature fusion strategy, which fuses features from different layers by means of the attention mechanism and layer-by-layer fusion. This strategy enables the model to better balance the information from different layers, enhances the expression of features, and improves the detection performance of the model. The gradient information during backpropagation is passed to the lower-level feature maps through jump connections to accelerate the gradient propagation and improve the convergence speed and training efficiency of the model.

BiFPN mainly realizes cross-layer information transfer and feature fusion. It has two main directions: one is upward convergence from lower feature layers, and the other is downward convergence from higher feature layers. In this way, targeted feature extraction can be performed on feature maps of different layers, and the size variation of the target at different scales can also be handled.

#### 2.3.4 ECA attention mechanism

ECA is a lightweight attention mechanism proposed by Wang et al. (2020). It is an attention model for computer vision tasks. It is mainly used to extract important information from image features for subsequent tasks such as image classification, target detection, and image segmentation. The core idea is to weigh and amplify the important feature channels and suppress the unimportant ones by performing attention operations on the channel dimension of the feature map.

**Figure 6:**
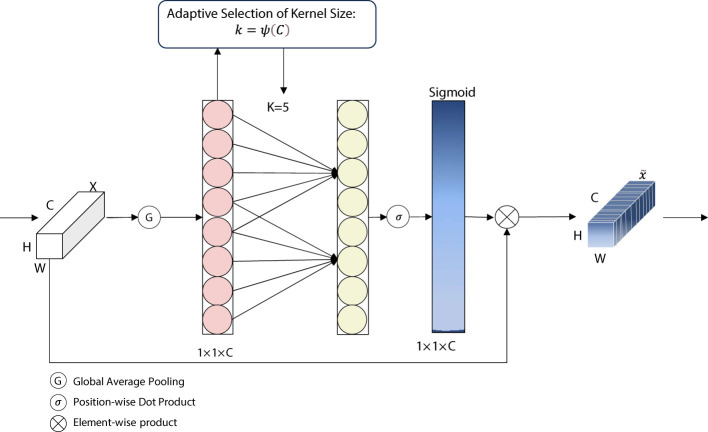
Diagram of ECA network structure. ECA, efficient channel attention.

ECA is a dimensionless module for implementing cross-channel interaction as shown in **Figure 6**. ECA implements a dimensionless cross-channel interaction. In this module, the input features are first compressed through a (GAP) layer, which in turn is sent into a one-dimensional (1D) convolutional layer for local channel interaction. This is then sent to the Sigmoid function, and this output is then multiplied by the input channel on an element-by-element, basis. The result of the product is the output of the ECA module. In this module, the size of the convolution kernel has a great impact on the sensory field while performing the convolution operation. If the size of the input feature map is large but a small convolution kernel is used, there is a possibility of losing some of the information, and vice versa. Therefore, the concept of dynamic convolution kernel is introduced into the ECA module, and the size of the convolution kernel can be dynamically selected according to the number of channels. ECA captures the local cross-channel interactions by considering each channel and its k nearest neighbors. ECA can be implemented by a fast one-dimensional convolution of size k, where k is the size of the kernel, which indicates the coverage of local cross-channel interactions, i.e., how many immediate neighbors are involved in the attentional prediction of a channel. k is determined using an adaptive determination method, where the kernel size k is proportional to the number of channel dimensions.

The ECA module introduces the feature of dynamic convolution kernels, which allows the selection of different sizes of convolution kernels depending on the number of channels. There exists a mapping relationship between the number of channels and the size of the convolution kernel as shown in Equation 1, and the exact mapping rules will vary depending on the specific implementation. This mechanism of dynamically selecting the size of the convolution kernel allows for the adaptive selection of the appropriate convolution kernel based on the characteristics of the input data, thus improving the performance and adaptability of the model.


(1) $$k=\psi \left(C\right)={\left|\frac{\log_{2}\left(C\right)}{\gamma }+\frac{b}{\gamma }\right|}_{odd}\;,\;$$

where *k* denotes the convolutional kernel size, *C* denotes the channel dimension, and *ψ* denotes the mapping relationship between *k* and *C*. The mapping relationship between *k* and *C* is shown in the following table. |t|odd denotes the closest odd number to t. *γ* and *b* are set to 2 and 1, respectively.

#### 2.3.5 GIOU loss function

GIOU loss function, proposed by Rezatofighi et al. (2019), is an excellent loss function for target detection tasks. It has the following advantages over other loss functions (e.g., the IOU loss function): first, GIOU is more accurate in terms of the precision of the target frame position and size, which is a better measure of the target detection accuracy. Second, GIOU can effectively prevent overlap and duplication between target frames during the training process, which promotes the stability and robustness of the target detection model. Finally, GIOU is relatively simple to compute, does not require the use of additional parameters or weights, is easy to implement and adjust, and can improve the performance and effectiveness of the target detection model.

The goal of GIOU is equivalent to adding a penalty to the loss function for the closure composed of ground truth and prediction frames, and its penalty term is the area of the closure minus the concatenation of the two frames in the closure that is as small as possible, which is able to more accurately assess the difference between the two bounding boxes. This makes it easier for the network to learn accurate bounding box predictions, leading to improved accuracy and robustness in target detection tasks.

The formula of GIOU is shown in Equation 2.


(2) $$GIOU_{loss}=1-GIOU=1-\left(IOU-\frac{C-A\cup^{}B}{C}\right)$$

## 3 Results

### 3.1 Experimental evaluation indicators

The configuration of the operating environment for this experiment included an operating system environment of Windows 11, a processor of 12th Gen Intel^®^ Core™ i5-12500 3.00 GHz, 32G of machine-banded operating memory, a 1TB SSD, and a graphics card of NVIDIA GeForce RTX 3080 with 10GB of video memory using GPU-accelerated computing. The software environment was as follows: Python 3.9, PyTorch 1.7.0, Torchvision 0.8.2, CUDA 11.0. The number of trial iterations was set to 400, the batch size was set to 8, and Adam was used as the optimizer. The initial learning rate of the model was set to 1e−3, the maximum learning rate was 1e−5, the momentum was 0.937, the weight decay was 0, and the input image resolution was set to 640 × 640. The same training parameters and dataset were used for all the models during the training process.

In order to evaluate the effectiveness of the network model in detecting the germination of wild rice seeds, the trained model was evaluated using the precision rate P (precision), the recall rate R (recall), the F1-score, and the mAP@0.5 (Abouelnaga et al., 2018; Kong et al., 2019; Huang et al., 2021; Mariusz and Marek, 2022) (mean average precision) as assessment metrics so as to validate and compare the performance of the model. Among them, precision rate P denotes the accuracy of the model in predicting the target, recall rate R denotes the success of the model in searching the target, and F1-score is the reconciled average of precision and recall, which is considered to be equally important, with the maximum of 1 and the minimum of 0. mAP@0.5 measures how good the model is in detecting all the categories. The definitions of precision rate P, recall rate R, F1-score, and mAP@0.5 are shown in Equations 3–6.


(3) $$\begin{array}{c} P=\frac{{\text{T}}_{P}}{{\text{T}}_{P}+{\text{F}}_{P}},\; \end{array}$$

(4) $$\begin{array}{c} R=\frac{{\text{T}}_{P}}{{\text{T}}_{P}+{\text{F}}_{N}},\; \end{array}$$

(5) $$\begin{array}{c} V_{F1-score}=2\times \frac{\text{P}\times \text{R}}{\text{P}+\text{R}},\; \end{array}$$

(6) $$\begin{array}{c} V_{mAP}=\frac{1}{C}\sum_{k=i}^{N}P\left(k\right)\Delta R\left(k\right) \end{array}$$

In the formula, TP, FP, and FN denote the number of true-positive, false-positive, and false-negative samples; VF1-score and VmAP represent the values of F1-score and mAP@0.5, respectively; C is the number of categories; N is the number of set thresholds; k is the set threshold; and P(k) and R(k) are the precision and recall, respectively, corresponding to the k value.

In identifying germinated seeds, germinated seeds are the identification target, TP denotes the number of seeds correctly identified as germinated seeds, FP denotes the number of seeds incorrectly identified as germinated seeds, and FN denotes the number of seeds incorrectly identified as ungerminated seeds, precision rate is the ratio of actual germinated seeds among all identified as germinated seeds, and recall rate is the ratio of germinated seeds identified among all actual germinated seeds. In identifying ungerminated seeds, ungerminated seeds are the identification target, TP indicates the number of seeds correctly identified as ungerminated seeds, FP indicates the number of seeds incorrectly identified as ungerminated seeds, FN indicates the number of seeds incorrectly identified as germinated seeds, the precision rate is the rate of actual ungerminated seeds among all identified ungerminated seeds, and the recall rate is the rate of actual ungerminated seeds among all ungerminated seeds that are recognized as ungerminated seeds. The recall rate is the percentage of ungerminated seeds identified out of all ungerminated seeds actually identified.

Germination rate is an important index for detecting seed germination, and in order to validate the germination detection results of this study, the germination rate of manual detection was taken and compared with the germination rate after SGR-YOLO discrimination. In addition, the germination potential, germination index (GI), and mean germination days (MGT) of the manual test were compared with the results of SGR-YOLO. Germination potential refers to the initial count of germination rate on day 3, and germination index and mean days to germination were calculated as shown in Equations 7, 8.


(7) $$\begin{array}{c} \;V_{GI}=\sum^{}\frac{{\text{G}}_{t}}{{\text{D}}_{t}},\; \end{array}$$

(8) $$\begin{array}{c} V_{MGT}=\frac{\sum^{}\left({\text{G}}_{t}+{\text{D}}_{t}\right)}{G} \end{array}$$

In Equations 7, 8, *V_GI_* is the value of the germination index, *V_MGT_* is the value of average days to germination, D*_t_* is the number of days to germination, G*_t_* is the number of newly germinated seeds per day corresponding to D*_t_*, and *G* is the germination rate (Zhang and Wang, 2021).

### 3.2 Analysis of test results of different attention mechanisms

In order to test the effect of different attention mechanism modules on the germination of wild rice seeds, the optimal attention mechanism was screened. The YOLOv7 model was tested on different attention mechanisms for comparison. Replacing different attention mechanisms, such as convolutional block attention module (CBAM) (Woo et al., 2018) attention mechanism, can help deep learning models to better focus and understand important features when processing images. Combining channel attention and spatial attention enables the model to adaptively select the most meaningful features for the current task, which improves the performance and robustness of the model. The global attention mechanism (GAM) helps the model to better understand the relevance of the input data and better capture the key information, which improves the performance and generalization ability of the model. Squeeze-and-excitation (SE) (Hu et al., 2018) attention mechanism is used to increase the model’s importance weights for different features to better learn and process data. It adaptively adjusts the weight of each feature, allowing the model to better focus on task-relevant features and improve model performance. Also included are ECA, Sim AM (A Simple, Parameter-Free Attention Module for Convolutional Neural Networks), and Selective Kernel (SK). The target detection model is trained without changing the parameters other than the backbone. The obtained parameters are analyzed and compared to verify the feasibility of the improved target detection model. The P, R, F1, and mAP@0.5 under different attention mechanisms are compared, and the results are shown in **Table 3**.

**Table 3:** Comparative analysis of the results of different attention mechanism network modules.

Training method	Network	Precision/%	Recall/%	F1/%	mAP/%
Hydroponic box	YOLOv7	87	91.7	86	90.9
	YOLOv7+CBAM	89.2	83.8	86	91.5
	YOLOv7+SK	89.8	89.9	86	90.6
	YOLOv7+GAM	89.4	88.7	89	88.6
	YOLOv7+SE	93.8	84.6	84	89.5
	YOLOv7+SimAM	89.6	89.1	85	89.4
	**YOLOv7>+ECA**	**86.9**	**87.3**	**89**	**92.2**
Petri dish	YOLOv7	95	96.4	81	95.4
	YOLOv7+CBAM	87.8	96.4	60	94.2
	YOLOv7+SK	90.4	97.2	79	95.3
	YOLOv7+GAM	88.5	95.6	64	94.4
	YOLOv7+SE	91.9	96.7	74	94.7
	YOLOv7+SimAM	91.4	97.3	76	95.4
	**YOLOv7+ECA**	**86.4**	**97.5**	**74**	**95.5**

The bold values indicates that it has the highest accuracy rate.

As can be seen from **Table 3**, the models, after the introduction of the attention mechanism, all have significant improvements in parameters. Among them, the average accuracy of the YOLOv7 network in the hydroponic box was 90.9%, and the average accuracy of the YOLOv7+ECA network was 92.2%, which was improved by 1.3 percentage points. The average accuracy of the YOLOv7 network under the Petri dish was 95.4%, and the average accuracy of the YOLOv7+ECA network was 95.5%, which was improved by 0.1 percentage points. The experimental results show that the introduction of the coordinate attention mechanism is effective for the extraction of wild rice germination in all cases, proving the effectiveness of adding the labeled attention mechanism in the improved version of YOLOv7.

**Figure 7:**
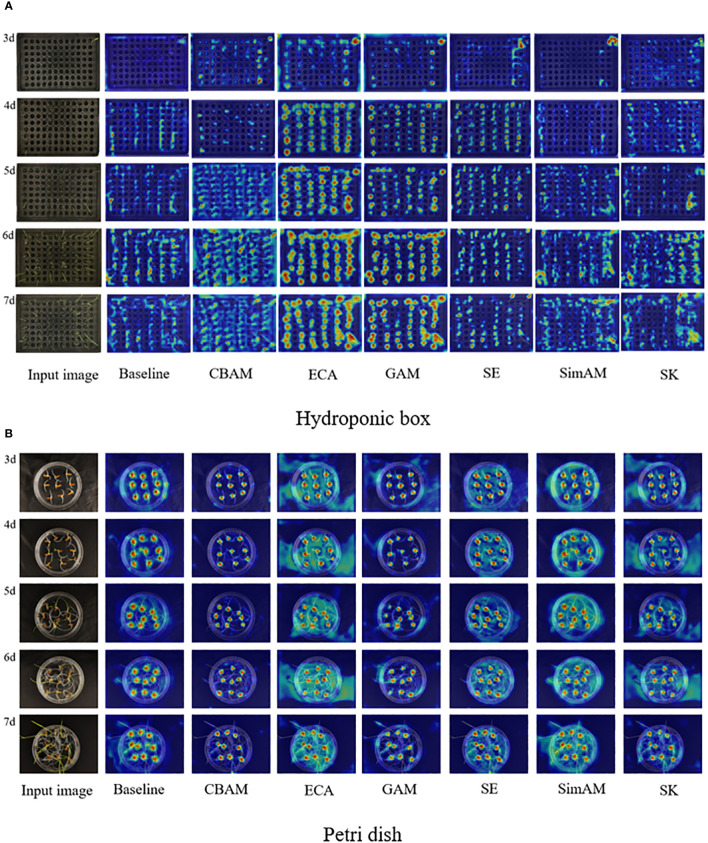
Heat map visualization results for different attention mechanism modules.

Heat map visualization of the improved YOLOv7 network detection process with the addition of six types of attention mechanisms is shown in **Figure 7**; after the addition of the attention mechanisms, the attention of each network becomes broad and deep, and the comparison of the heat maps under different days shows that ECA has the best effect. Under the thermodynamic effect of the hydroponic box (**Figure 7A**), the GAM attention mechanism is slightly worse than ECA, the hydroponic box is denser relative to the Petri dish, and ECA is more effective compared with SimAM, CBAM, SK, and SE attention mechanisms. Under the thermodynamic effect of the Petri dish (**Figure 7B**), SK and SE are slightly less effective compared with ECA, SimAM, CBAM, and GAM, and ECA is more effective. The experimental results and visualization show that adding the ECA attention mechanism to the network model can effectively improve the detection accuracy of the overall network.

### 3.3 Experimental results with different loss functions

In order to test the effect of different loss functions on the germination of wild rice seeds, the optimal loss functions were screened and compared with the loss functions tested on the YOLOv7 model. The replacement of different loss functions, such as CIOU, is based on DIOU to increase the loss of the detection box scale and increase the length and width of the loss so that the prediction box will be more in line with the real box. DIOU based on the IOU characteristics, takes into account the shortcomings of the shores of mooching GIOU, a direct regression on the Euclidean distance of the center point of the two frames, to accelerate the convergence. EIOU, on the basis of the CIOU, calculates the difference value of width and height to replace the aspect ratio, and at the same time, Focal Loss is introduced to solve the problem of imbalance between difficult and easy samples. GIOU introduces the minimum outer frame label on the basis of IOU characteristics to solve the problem of loss equal to 0 when the detection frame and the real frame do not overlap. SIOU, as well as Focal, trains the target detection model without changing the parameters other than the main stem. The obtained parameters are analyzed and compared to verify the feasibility of the improved target detection model. P, R, F1, and mAP@0.5 are compared under different loss functions.

The effects of different loss functions on the overall performance of the model were analyzed and compared. By replacing different types of bounding box regression loss functions, the prediction box direction drift is improved, and the convergence speed and detection performance of the model are improved. The results are shown in **Table 4**, which shows that SGR-YOLO, after using GIOU, is significantly improved in all parameters. Among them, the average accuracy of the YOLOv7 network under the hydroponic box was 90.9%, and the average accuracy of the YOLOv7+ECA network was 91.5%, which was improved by 0.6 percentage points. The average accuracy of the YOLOv7 network under the Petri dish was 95.4%, and the average accuracy of the YOLOv7+ECA network was 95.6%, which was improved by 0.2 percentage points. GIOU takes the size of the box into account when calculating the overlap degree, which can more accurately measure the overlap of the box and improve the overall performance of the model.

**Table 4:** Comparison of different loss functions.

Training method	Network	Precision/%	Recall/%	F_1_/%	mAP/%
Hydroponic box	YOLOv7	87	88.9	86	90.9
	YOLOv7+CIOU	88.6	84.4	86	91.1
	YOLOv7+DIOU	88.2	84.2	86	90.8
	YOLOv7+EIOU	86.6	83.1	85	89.6
	YOLOv7+SIOU	87.9	84.3	86	90.8
	YOLOv7+Focal	86.3	84.8	86	90.1
	YOLOv7+**GIOU**	**88.6**	**84.8**	**87**	**91.5**
Petri dish	YOLOv7	95	96.4	81	95.4
	YOLOv7+CIOU	94.5	96.4	82	94.8
	YOLOv7+DIOU	92.6	96.9	76	95.2
	YOLOv7+EIOU	81.9	92.4	60	89.5
	YOLOv7+SIOU	91.1	96.9	76	94.7
	YOLOv7+Focal	93.5	96.9	76	95
	YOLOv7+**GIOU**	**94.3**	**96.5**	**79**	**95.6**

The bold values indicates that it has the highest accuracy rate.

### 3.4 Ablation experiments

Ablation experiments were performed on the constructed dataset, and the results are shown in **Table 5**. YOLOv7 was used as the benchmark model in this experiment. BiFPN structure was adopted in YOLOv7, ECA attention was added in YOLOv7, and the GIOU function was used as a loss function as a way to verify the significance of each module. In the hydroponic box experiment, we found that after adding BiFPN, compared to the initial YOLOv7 model, mAP@0.5 increased by 0.7%. After incorporating the ECA attention mechanism, mAP@0.5 increased by 1.3%. When replacing the loss function with GIOU, mAP@0.5 increased by 0.6%. After using BiFPN together with the ECA module, mAP@0.5 increased by 2.4%. After using BiFPN together with GIOU, mAP@0.5 increased by 2.5%. After using ECA together with GIOU, mAP@0.5 increased by 2.2%. After using BiFPN, ECA, and GIOU together, mAP@0.5 increased by 3.1%. We found that after adding BiFPN, compared to the initial YOLOv7 model, mAP@0.5 increased by 2.3%. After incorporating the ECA attention mechanism, mAP@0.5 increased by 0.1%. After replacing the loss function with GIOU, mAP@0.5 increased by 0.2%. After using BiFPN together with the ECA module, mAP@0.5 increased by 1.7%. After using BiFPN together with GIOU, mAP@0.5 increased by 2.1%. After using ECA together with GIOU, mAP@0.5 increased by 2.5%. After using BiFPN, ECA, and GIOU together, mAP@0.5 increased by 2.8%. All analyses show that the improved model outperforms other models. It has good real-time performance and greatly improves the detection of small targets.

**Table 5:** Results of ablation experiments for the model.

Training method	Network	Precision/%	Recall/%	F_1_/%	mAP/%
Hydroponic box	YOLOv7	87	88.9	86	90.9
	YOLOv7+BiFPN	89	86	88	91.6
	YOLOv7+ECA	86.9	87.3	89	92.2
	YOLOv7+GIOU	88.6	84.8	87	91.5
	YOLOv7+BiFPN+ECA	88.2	89.8	89	93.3
	YOLOv7+BiFPN+GIOU	90.4	90.9	91	93.4
	YOLOv7+ECA+GIOU	91.1	87	89	93.1
	YOLOv7+BiFPN+ECA+GIOU	91.1	88.9	90	94
Petri dish	YOLOv7	95	96.4	81	95.4
	YOLOv7+BiFPN	90.4	98.1	81	97.7
	YOLOv7+ECA	86.4	97.5	74	95.5
	YOLOv7+GIOU	94.3	96.5	79	95.6
	YOLOv7+BiFPN+ECA	88.9	98.1	73	97.1
	YOLOv7+BiFPN+GIOU	88.9	98.8	80	97.5
	YOLOv7+ECA+GIOU	91.5	98.1	85	97.9
	YOLOv7+BiFPN+ECA+GIOU	95.1	97.2	87	98.2

### 3.5 Comparative tests

In order to evaluate the SGR-YOLO model, under the same experimental conditions, the accuracy P, recall R, harmonic mean F1, and average accuracy were measured. mAP@0.5 of YOLOv5 was compared with YOLOv7 in four aspects. The results are shown in **Table 6**. The detection performance of the three networks varies. The SGR-YOLO model has certain advantages in terms of accuracy and recall. The improved model adds the BiFPN structure, which makes the overall model more accurate for the small target detection layer.

**Table 6:** Comparison of detection performance of different networks.

Training method	Model	Precision/%	Recall/%	F_1_/%	mAP/%
Hydroponic box	YOLOv5	73.7	74.4	74	64.8
	YOLOv7	87	88.9	86	90.9
	SGR-YOLO	91.1	91.7	90	94
Petri dish	YOLOv5	85.9	73	78	78.6
	YOLOv7	95	96.4	81	95.4
	SGR-YOLO	95.1	97.2	87	98.2

The SGR-YOLO network structure proposed in this article was applied to the water culture box, with mAP@0.5 of 94%, and the culture dish, with mAP@0.5 of 98.2%. Compared to the original YOLOv5 and YOLOv7 models, the water culture box increased by 29.8% and 3.1%, respectively, and the plate increased by 19.6% and 2.8%, respectively.

The other values of the SGR-YOLO network structure were higher than the corresponding indexes of the other two models, which proves the effectiveness of the SGR-YOLO network structure in the detection of the germination rate of wild rice seeds.

### 3.6 Results of wild rice seed germination rate test

**Figure 8:**
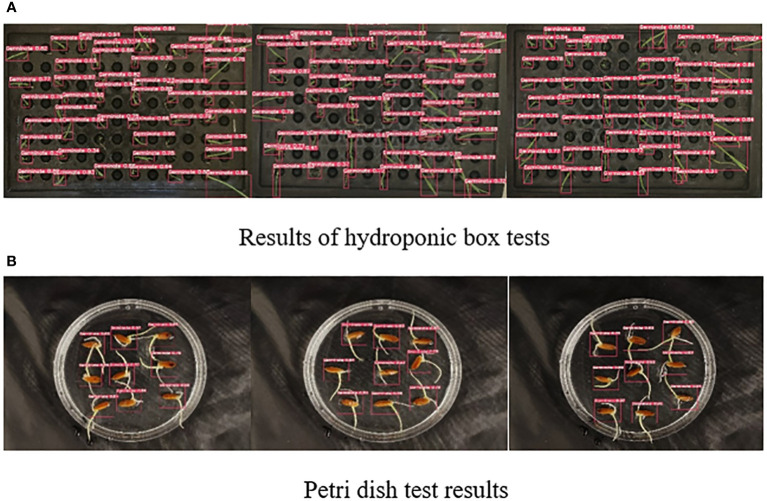
Results of SGR-YOLO seed germination test.

In order to evaluate the performance of the model more accurately, we randomly selected some images for wild rice seed germination detection. **Figure 8A** demonstrates the detection results of the hydroponic box. During the germination process of wild rice, the leaves overlapped and crossed over as time passed, and the size of the leaves varied due to different growth rates. The SGR-YOLO assay performed very well in the face of these complex conditions. However, **Figure 8B** shows the assay results of Petri dishes. In the Petri dishes, the buds and roots of the seeds grew at the same time, resulting in a complex background. The SGR-YOLO assay still performed well under this complex background.

The germination rates of the seeds in the test set were compared between manual detection and the SGR-YOLO model. The seeds did not germinate on days 0 and 1, started germinating on day 2, reached the peak germination period on day 3, with most seeds having germinated, and the germination rate stabilized by day 5. In the hydroponic box, the germination rate of the seeds gradually increased from day 1 to day 7. On day 1, the seeds had not germinated. On day 2, the manual detection reported a germination rate of 31.5%, while SGR-YOLO detected 28.5%. On day 3, the manual detection reported a germination rate of 86.3%, while SGR-YOLO detected 85%. By day 5, the manual detection reported a germination rate of 91%, while SGR-YOLO detected 90.5%. In the petri dish, on day 1, the seeds had not germinated. On day 2, the manual detection reported a germination rate of 36.2%, while SGR-YOLO detected 35.7%. On day 3, the manual detection reported a germination rate of 87.3%, while SGR-YOLO detected 84%. The germination rate stabilized by day 5, with the manual detection reporting a rate of 98.4% and SGR-YOLO detecting 97.4%. Due to simultaneous growth of roots and seedlings in the petri dish, SGR-YOLO occasionally recognized them as separate entities, resulting in a slightly lower germination rate during detection.

Combined with the detection model, the 7-day germination data of the test set were counted, and the seed germination rate, germination potential, germination index, average days to germination, and calculation time were measured. The results are shown in **Table 7**. The discrepancies between SGR-YOLO and manual detection in terms of germination vigor, germination index, and average germination days in the hydroponic box were 0.4%, 2.2, and 0.9 days. For the petri dish detection, the disparities in germination vigor, germination index, and average germination days between SGR-YOLO and manual detection were 0.5%, 0.5, and 0.24 days. In the hydroponic boxes, the time for manual detection of the test set images was 3,850 s, while SGR-YOLO needed only 32.46 s. In Petri dishes, the time for manual detection of the test set images was 1,920 s, while SGR-YOLO needed only 32.98 s. The SGR-YOLO significantly reduces the detection time by eliminating the need for manual individual processing, whereas manual detection requires processing each item one by one, resulting in lower efficiency. SGR-YOLO can be used to discriminate wild rice seed germination and calculate seed germination rate.

**Table 7:** Comparison of seed germination test indicators.

Training method	Means	Germination rate/%	Germination potential/%	Germination index	Average days to germination/days	Computation time/s
Hydroponic box	Manual detection	91.6	83.4	68.83	2.56	3850
	SGR-YOLO	91.2	83	67.93	2.67	32.46
Petri dish	Manual detection	98.4	86.5	73.87	2.12	1920
	SGR-YOLO	97.4	86	72.76	2.36	32.98

## 4 Discussion

In order to solve the problem of seed germination rate detection, Jin et al. (2022) used the full spectrum and feature wavelengths selected by principal component analysis (PCA) to construct a convolutional neural network (CNN) and traditional machine learning methods (support vector machine (SVM) and logistic regression (LR)) for predicting the vigor of different varieties of rice seeds under natural aging conditions. The accuracy of most models was above 85%. Peng et al. (2022) designed the DDST-Center Net algorithm proposed for the automatic monitoring system of seed germination test. The algorithm is computationally efficient, but it is only applicable to seed germination of oilseed rape. Wang et al. (2021). proposed a non-destructive monitoring method for the growth process of cucumber seedlings based on a Kinect camera, which carried out non-destructive monitoring of the germination rate, plant height, leaf area, and other parameters of cucumber seedlings, and the germination rate error was not more than 1.567%. These methods all use machine vision technology to extract germination features and realize seed germination discrimination through morphological detection, but the seed germination characteristics of different crops are different, which leads to the limitations of the application of these methods.

This paper proposed a model method based on a deep learning model to detect the seed germination rate of wild rice. First, dynamic image collection was carried out on two different germination methods, Petri dish and hydroponic box; then, the comparative test of different attention mechanisms and loss functions was carried out, among which the ECA attention mechanism and GIOU function had the best effect, and then the ablation test was carried out. Finally, the improved model SGR-YOLO was used to analyze the germination rate of wild rice seeds, and the following conclusions were drawn.

1. The SGR-YOLO model adds the ECA attention mechanism to the backbone to focus on the feature differences, reduces the data dimension, and improves the accuracy and speed; after the BiFPN structure is introduced in the Neck part, the computational efficiency of the model is improved, but the accuracy is not significantly improved; the detection accuracy is improved by improving the loss function in the Prediction part.
2. By comparing the results of different attention mechanisms, it was found that the ECA attention mechanism has the best effect, the heat map under different days shows that the ECA has the best effect, and the attention of each network becomes broad and deep. Comparing the results of different loss functions, the GIOU function has the best effect. First, when the BiFPN structure was introduced into the hydroponic box, the accuracy rate reached 91.6%, and that of the Petri dish reached 97.7%; then, the ECA attention mechanism and GIOU function were added, and the mAP@0.5 of the model in the hydroponic box was 94%, which was increased by 3.1% compared with the YOLOv7 model, and the mAP@0.5 in the Petri dish was 98.2%, which was 2.8% higher than that of the YOLOv7 model. Compared with the YOLOv5 and YOLOv7 models, the SGR-YOLO model is better than the comparison model in terms of accuracy, recall, F1 value, parameter quantity, and computational cost.
3. In order to further optimize SGR-YOLO, a comparative test was carried out on SGR-YOLO, and the error between the germination rate of SGR-YOLO and the manual detection in the hydroponic box was 0.4% considering the seed germination rate, germination potential, germination index, average germination days, and calculation time. The discrepancy between SGR-YOLO and manual detection in terms of germination rate in the petri dish was 1%. In the hydroponic box, the image time of the manual test set was 3,850 s, and the image time of SGR-YOLO only needed 32.46 s; in the Petri dish, the image time of the manual test set was 1,920 s, and the image time of SGR-YOLO only needed 32.98 s.
4. The SGR-YOLO model can calculate the seed germination rate more intuitively and quickly through multi-day image combination detection, which provides a feasible method for automatic seed germination detection. This research method has certain accuracy and scientificity, which lays a research foundation for the subsequent intelligent identification of the germination rate of wild rice seeds and also provides a certain theoretical basis and reference value for target detection and recognition using images obtained by mobile phones. Seed germination models can be used to assess the quality and vigor of seeds. By predicting the germination rate and germination time of seeds, it is possible to judge the health and viability of seeds. It can help select high-quality seeds and improve the yield and quality of crops.

## Data availability statement

The raw data supporting the conclusions of this article will be made available by the authors, without undue reservation.

## Author contributions

QY: Writing – original draft. XZ: Writing – review & editing. GZ: Writing – review & editing. JZ: Writing – review & editing.
